# Genomic duplication problems for unrooted gene trees

**DOI:** 10.1186/s12864-015-2308-4

**Published:** 2016-01-11

**Authors:** Jarosław Paszek, Paweł Górecki

**Affiliations:** University of Warsaw, Institute of Informatics, Banacha 2, Warsaw, 02-097 Poland

**Keywords:** Genomic duplication, Duplication episode, Reconciliation, Unrooted gene tree, Species tree

## Abstract

**Background:**

Discovering the location of gene duplications and multiple gene duplication episodes is a fundamental issue in evolutionary molecular biology. The problem introduced by Guigó et al. in 1996 is to map gene duplication events from a collection of rooted, binary gene family trees onto theirs corresponding rooted binary species tree in such a way that the total number of multiple gene duplication episodes is minimized. There are several models in the literature that specify how gene duplications from gene families can be interpreted as one duplication episode. However, in all duplication episode problems gene trees are rooted. This restriction limits the applicability, since unrooted gene family trees are frequently inferred by phylogenetic methods.

**Results:**

In this article we show the first solution to the open problem of episode clustering where the input gene family trees are unrooted. In particular, by using theoretical properties of unrooted reconciliation, we show an efficient algorithm that reduces this problem into the episode clustering problems defined for rooted trees. We show theoretical properties of the reduction algorithm and evaluation of empirical datasets.

**Conclusions:**

We provided algorithms and tools that were successfully applied to several empirical datasets. In particular, our comparative study shows that we can improve known results on genomic duplication inference from real datasets.

## Background

Genomic duplication plays important role in evolution of life on Earth. This phenomenon have been extensively studied in the last decades for plant, bacterial and many other genomes [1–7]. Duplication events can involve individual genes, genomic segments or whole genomes. While the reconstruction of evolutionary history of individual genes is generally well established [8–13], still little is known on the inference of large genomic duplications that can span through thousands of genes families.

In this approach we propose to use the model of reconciliation in which a gene tree is reconciled with its species tree. The concept of reconciliation was introduced by Goodman [14] and formalized by Page [8] in the context of reconciling potential incongruence between a rooted gene family tree and its species tree. In this model, differences between gene and species trees are explained in terms of evolutionary events such as gene duplication, gene loss and speciation. Reconciliation can be interpreted as the embedding of a gene tree into a species tree where these evolutionary events, located in the species tree, induce a biologically consistent scenario [15]. Tree reconciliation has been extensively studied in recent decades in many theoretical and practical contexts including supertree inference, error correction and HGT detection [16–24]. In the process of reconciliation, which is relatively simple from computational point of view, each gene from a single gene family is mapped into the species tree and it is classified as a single gene duplication or related to speciation. However, the problem becomes much more complex, when a gene duplication is a part of large genomic duplications, called *multiple gene duplication episode*, in which parts of a genome are duplicated. In fact, it is known that a large duplication event is usually followed by many gene losses and gene rearrangements. In consequence, the reconstruction of large gene duplication events may be difficult.

The first approach to detect multiple gene duplication episodes from a collection of rooted gene trees was proposed by Guigó et al. [10]. In the model, for a given collection of rooted gene trees and a rooted species tree, the authors proposed heuristic to aggregate single gene duplication events into a large gene duplication. This approach was formalized and refined by Page and Cotton [25]. They formally defined the problem of *episode clustering* (EC) as the problem of locating the minimal number of locations in the species tree, where all duplications from the input gene trees can be placed. This model was applied in the context of the supertree problem by Fellows [26]. Burleigh et al. [27] and Bansal and Eulenstein [28] proposed the first polynomial time solutions for two types of the multiple gene duplication problems: the episode clustering (EC) and a more general variant of clustering called *minimum episodes* (ME). Finally, Luo et al. [29] proposed linear time and space algorithms to these problems.

While the classical reconciliation model is applicable to rooted trees only, most standard phylogenetic inference methods, like maximum likelihood, maximum parsimony or neighbour joining, infer unrooted gene family trees, and it is often difficult, to identify credible rootings. For example, outgroup rooting can result in incorrect rootings when evolutionary events cause heterogeneity in the gene trees, and rooting gene trees under the molecular clock assumption, or similarly by using midpoint rooting, also can result in error when there is a molecular rate variation throughout the tree [30, 31]. Tree reconciliation have been successfully extended to reconcile an unrooted gene tree with a rooted species tree by seeking a rooting of the unrooted gene tree that invokes the minimum number of evolutionary events such as gene duplications (D) or gene duplications and losses (DL), in the context of a given species tree [32, 33]. It is known that the rooting edges with minimal D or DL cost, induce a full subtree, called *plateau*, in the unrooted gene tree [34].

In this article we present the first solution to the open problem [27] of *unrooted episode clustering*, that is, the problem of episode clustering where the input consists of unrooted gene trees. We show that for a given set of unrooted gene trees and a species tree we can solve the unrooted episode clustering by reducing it to the rooted episode clustering problem that has a linear time complexity. Our solutions require a linear time preprocessing and a creation of at most 1+2^*k*^ collections of rooted gene trees, that is, instances of rooted EC Problem, where *k* is the number of input gene trees having a special topology located in the plateau of the duplication cost (formally, the condition requires two stars S2 [32]). Usually *k* represents a small fraction of the whole input, thus, this condition significantly reduces the complexity. In other words, we show that the problem of unrooted episode clustering is fixed parameter tractable. Finally, in a number of empirical computational experiments we show that despite the exponential worst case complexity our algorithm is able to resolve instances of the problem after the verification of at most two rooted datasets. In consequence, our solution can be efficiently applied to locate duplication clusters in collections of unrooted gene trees.

## Results

### Basic notation

A *species tree* is a rooted binary tree with leaves uniquely labeled by the names of species. Throughout this work, the species tree is fixed, therefore, we use *S* to denote it. A *rooted gene tree* is a rooted binary tree with leaves labeled by the names of species. The set of species present in *T* is denoted by $\mathcal {L}(T)$. The rooted tree (*T*_1_,*T*_2_) has two subtrees *T*_1_ and *T*_2_ whose roots are children of the tree root. Additionally, for nodes *a* and *b*, *a*≼*b* means that *a* and *b* are on the same path from the root, with *b* being closer to the root than *a*. We write *a*≺*b* if *a*≼*b* and *a*≠*b*. The root of a tree *T* we denote by *root*(*T*).

**Fig. 1:**
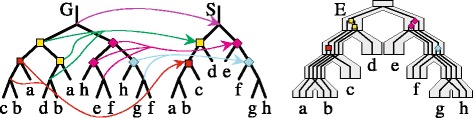
Gene and species tree reconciliation. *Left*: the lca-mapping between a gene tree *G* and a species tree *S* shown for internal nodes. The decoration of nodes indicates gene duplication events. *Right*: an embedding of *G* into *S*. To reconcile these trees 6 gene duplications and 17 gene losses (not shown) are required, i.e., D(*G,S*)=6 and *DL*(*G,S*)=23. See also *G*_4∗_ in Fig. 3

Let *T*=〈*V*_*T*_,*E*_*T*_〉 be a rooted gene tree such that $\mathcal {L}(T) \subseteq \mathcal {L}(S)$. *The least common ancestor (lca) mapping*, M_*T*_:*V*_*T*_→*V*_*S*_, is defined as follows. If *v* is a leaf in *T* then M_*T*_(*v*) is the leaf in *S* labeled by the label of *v*. When *v* is an internal node in *T* having two children *a* and *b*, then M_*T*_(*v*) is the least common ancestor of M_*T*_(*a*) and M_*T*_(*b*) in *S*. An internal node *g*∈*V*_*T*_ is called a *duplication* if M_*T*_(*g*)=M_*T*_(*a*) for a child *a* of *g*. *The duplication cost*, denoted by D(*T,S*), is the total number of duplications in *T*. Each non-duplication node of *T* we call a *speciation*. The total number of *gene losses* required to reconcile *T* and *S* can be defined by: $\mathsf {L}(\textit {T,S})=2\mathsf {D}(\textit {T,S})+\sum _{g\ \text {is internal}, \textit {a,b}\ \text {children of}\ g} (\|\mathsf {M}_{T}(a),\mathsf {M}_{T}(b)\|-2)$, where ∥*a,b*∥ is the number of edges on the path connecting *a* and *b* in *S*. Finally, we can define the *duplication-loss cost* of reconciling a rooted gene tree *T* and a species tree *S* as follows: *DL*(*T,S*)=D(*T,S*)+L(*T,S*) [34]. Examples of the reconciliation are depicted in Fig. 1.

### Unrooted reconciliation

*The unrooted gene tree* is an undirected acyclic connected graph in which each node has degree 1 (leaves) or 3 (internal nodes), and the leaves are labeled by the names of species. For an unrooted gene tree *G*=〈*V*_*G*_,*E*_*G*_〉 and an edge *e*∈*E*_*G*_, by *G*_*e*_, we denote the rooting of *G* obtained from *G* by placing the root on *e*. Such a rooting induces the duplication cost D(*G*_*e*_,*S*). We call *D-minimal*, the rooting or edges having the minimal duplication cost. It follows from the theory of unrooted reconciliation [32, 34] that the set of D-minimal edges, called *D-plateau*, is a full subtree of *G*. The same property holds for the DL-plateau, that is, the set of edges with the minimal duplication-loss cost. We use a similar notation for DL-minimal edges, rootings and so on. The most important property of these plateaus is below.

#### **Theorem1** (From [34]).

DL-plateau is a subgraph of D-plateau.

Without loss of generality we assume that every root of a gene tree is mapped into the root of *S*, denoted by ⊤, and both trees are non-trivial. An edge *e*=〈*v,w*〉 of *G* is *empty* if the root of *G*_*e*_ is a speciation, i.e., $\mathsf {M}_{G_{e}}(v) \neq \top \neq \mathsf {M}_{G_{e}}(w)$. We call *edouble* if $\mathsf {M}_{G_{e}}(v)=\top =\mathsf {M}_{G_{e}}(w)$. Otherwise, *e* is called *single*. A single edge *e* is called *v*-incoming or *w-outgoing* if $\mathsf {M}_{G_{e}}(v) \neq \top = \mathsf {M}_{G_{e}}(w)$.

**Fig. 2:**
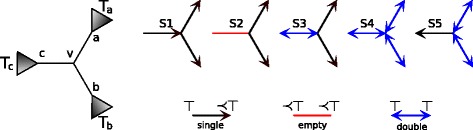
Types of stars. Star topology with the center *v*, types of edges and stars

Let *v* be an internal node of *G*, then a *star* with a *centerv* consists of three edges, denoted by *e*_*a*_, *e*_*b*_ and *e*_*c*_, sharing *v* and incident to nodes *a*, *b* and *c*, respectively (see Fig. 2). The are several types of possible star topologies based on the above classification of edges: the *S*1 star has one *v*-incoming edge and two *v*-outgoing edges, the *S*2 star has exactly two *v*-outgoing edges and one empty edge, the *S*3 star has two *v*-outgoing edges and one double edge, the *S*4 star all 3 edges are double, and the *S*5 star has one *v*-outgoing edge and two double edges. The star topologies are depicted in Fig. 2.

#### **Theorem2** (Adopted from [32]).

For a given unrooted gene tree *G*, we have

- either *G* has exactly one empty edge or *G* has at least one double edge,
- if the DL-plateau of *G* consists of exactly one edge, then this edge is either empty or double, and all other edges are single.
- if the DL-plateau of *G* has more than one edge, then it contains all edges present in stars *S*4 and *S*5, and all other edges are single.

**Fig. 3:**
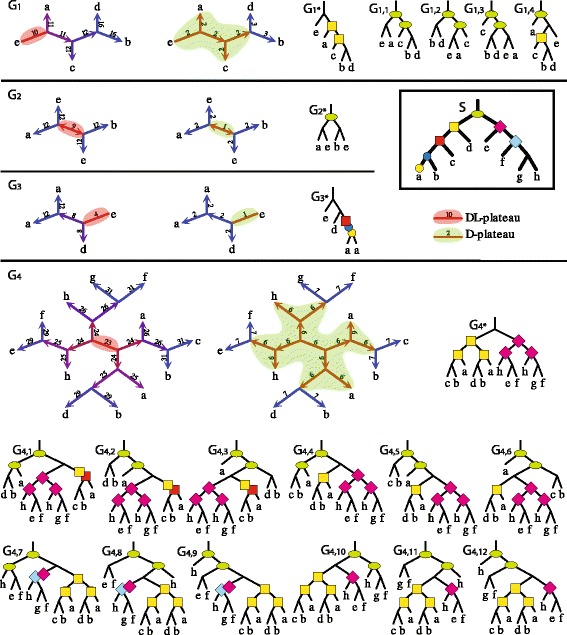
An example of unrooted episode clustering. A species tree *S* and four unrooted gene trees *G*_1_, *G*_2_, *G*_3_, *G*_4_ with all D-minimal rootings. For every gene tree two star topologies are shown: one for the duplication-loss cost (*left*) and one for the duplications cost (*right*). Every edge of a gene tree is decorated with the corresponding cost of rooting. Every duplication node in rootings of gene trees is decorated by all possible locations (i.e., valid mappings) of its duplication cluster from optimal solutions of single-UEC. Note that the rooting *G*_4∗_, whose lca-mappings are shown in Fig. 1, has two duplications at (*c*,(*b,a*)) and (*h*,(*f,g*)) that are raised (here) to create two duplications clusters. Let {*G*_2_,*G*_4_} be an instance of UEC Problem. Then, the ⊤-cluster, that is present in *G*_2∗_, contributes to the optimal solution. In such a case, the solution is induced by one of the two instances of EC problem: {*G*_2∗_,*G*_4,1_} or {*G*_2∗_,*G*_4,7_}. This property is proved in Theorem 5 and in Lemma 6

Note that if a gene has an empty edge, then it has at most two stars S2 (see examples in Fig. 3).

### Episode clustering problems

To model gene duplication episodes we allow to relocate a gene duplication from its lca-mapping location to one of its ancestors. In other words, we introduce mappings representing evolutionary scenarios that can differ from the scenario defined by the lca-mapping. Additionally, we require that the total number of gene duplications is minimal. To ensure biological correctness of such mappings, we introduce several conditions, e.g., time order preservation.

A mapping F_*G*_:*V*_*G*_→*V*_*S*_ is called *valid* if the following conditions are satisfied:

- F_*G*_(*a*)≼F_*G*_(*b*) if *a*≼*b* (time consistency),
- F_*G*_(*a*)=M_*G*_(*a*) for any speciation node *a* (fixed speciations),
- F_*G*_(*a*)≽M_*G*_(*a*) for any duplication node *a* (duplication can be raised),
- F_*G*_(*a*)≺M_*G*_(*b*) for any speciation node *b* such that *a*≺*b* (fixed number of gene duplications).

It can be shown that every valid mapping uniquely defines an evolutionary scenario represented by a DLS-tree [15]. Additionally, every DLS-tree obtained from a valid mapping can be transformed into the optimal evolutionary scenario (i.e., lca-based scenario), by a sequence of TMOVE (i.e., lowering duplication) transformations. Please refer to [15] for more details on formal modeling of evolutionary scenarios. Observe, that the above model is more general than the model from [28].

We denote by *Dup*(*T*), the set of all duplication nodes in *T*. Let *G*_1_,*G*_2_,…,*G*_*n*_ be a collection of rooted gene trees. Assume that, for every *i*∈{1,2,…,*n*}, F_*i*_ is a valid mapping between *G*_*i*_ and the species tree *S*. Every element $s \in \bigcup _{i} \mathsf {F}_{i}(\mathsf {Dup}_{G_{i}})$ denotes the location of multiple gene duplication events in *S*. Such locations will be called *duplication episodes*. A *duplication cluster* for *s* is the set of all gene duplications present in *G*_*i*_’s that are mapped to *s*. By ⊤-cluster we denote the duplication cluster whose elements are mapped to ⊤.

#### **Problem1** (Rooted Episode Clustering (EC)).

Given a collection of rooted gene trees *G*_1_,*G*_2_,…,*G*_*n*_ and a species tree *S*. Compute the minimal number of duplication episodes, denoted by *EC*(*G*_1_,*G*_2_,…,*G*_*n*_,*S*), in the set of all valid mappings F_1_,F_2_,…,F_*n*_ such that $\mathsf {F}_{i} \colon V_{G_{i}} \rightarrow V_{S}$.

This problem can be solved in linear-time and space [29]. In this article we solve the following problem.

#### **Problem2** (Unrooted Episode Clustering (UEC)).

Given a collection of unrooted gene trees *G*_1_,*G*_2_,…,*G*_*n*_ and a species tree *S*. Compute the minimal *EC*(*T*_1_,*T*_2_,…,*T*_*n*_,*S*) in the set of rooted gene trees {*T*_1_,*T*_2_,…,*T*_*n*_} such that *T*_*i*_ is a rooting obtained from *G*_*i*_ by placing the root on the edge from the D-plateau.

Observe, that we allow rootings only in the D-plateau. Otherwise, the total number of gene duplications is not minimal. By single-UEC we denote the problem UEC for a single unrooted gene tree, i.e., when *n*=1. Every edge in an unrooted gene tree that induces the optimal solution for single-UEC will be called *optimal* (for single-UEC). For convenience, we use *EC*(*T*_1_,*T*_2_,…,*T*_*n*_) instead of *EC*(*T*_1_,*T*_2_,…,*T*_*n*_,*S*).

### Episodes in a gene tree with an empty edge

In this Section we solve single-UEC problem for the case when the input gene tree has one empty edge.

Let *v* be a center of the star that contains the only DL-plateau edge in a gene tree *G*. This star induces three rooted subtrees *T*_*a*_, *T*_*b*_ and *T*_*c*_ rooted at neighbours *a*, *b* and *c*, respectively, as indicated in Fig. 2. Let  be the indicator function, that is,  is 1 if *p* is satisfied and 0 otherwise.

#### **Lemma1**.

**Fig. 4:**
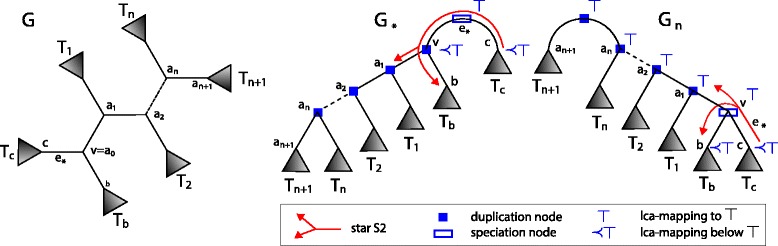
Trees from Lemma 1 and 2. A gene tree *G* (*left*) and the rootings of *G* (*right*) from Lemma 1 and Lemma 2

Let *a*_0_,*a*_1_,*a*_2_,…,*a*_*n*+1_ (for *n*≥0) be the path of D-plateau nodes connecting *v*=*a*_0_ and *a*_*n*+1_∈*T*_*a*_ in *G*. Let *G*_*n*_ be the D-minimal rooting induced by the edge 〈*a*_*n*_,*a*_*n*+1_〉. If *e*_∗_=〈*v,c*〉 is empty then



where *T*_1_,*T*_2_,…,*T*_*n*+1_ are subtrees of *T*_*a*_ such that *T*_*a*_=(*T*_1_,(*T*_2_,…,(*T*_*n*_,*T*_*n*+1_)…)) and the root of *T*_*n*+1_ is *a*_*n*+1_ (see Figs. 2 and 4).

#### *Proof*.

First we show that *v* is a speciation node in *G*_*n*_. It follows from the fact that *v* is a center of S2 star and 〈*v, b*〉 is single. Thus, M_*n*_(*v*)=⊤, M_*n*_(*c*)≺⊤ and M_*n*_(*b*)≺⊤, where M_*n*_ is the lca-mapping for *G*_*n*_. From the fact that M_*n*_(*v*)=⊤ we conclude that all nodes on the path connecting the parent of *v* with the root in *G*_*n*_ are mapped to ⊤, therefore, they are duplications.

Lets consider the number of duplication clusters in *G*_*n*_. We have the ⊤-cluster composed of the duplication nodes *a*_1_,*a*_2_,…,*a*_*n*_,*root*(*G*_*n*_) mapped to ⊤. Both *T*_*c*_ and *T*_*b*_ in *G*_*n*_ are under speciation node *v* so their clusters are disjoint with the ⊤-cluster. Finally, if the root of some *T*_*i*_ is a duplication then its cluster can be merged with the ⊤-cluster. Therefore, the ⊤-cluster contributes to *EC*(*G*_*n*_) only if the root of *T*_*i*_ is a speciation for every *i*. Now, it is easy to conclude the final formula.

#### **Lemma2**.

Under the assumptions from the previous lemma, we have



where *G*_∗_ is the rooting induced the empty edge *e*_∗_=〈*v,c*〉 (see Fig. 4).

#### *Proof*.

Both rootings *G*_*n*_ and *G*_∗_ are D-minimal. Hence, D(*G*_∗_,*S*)=D(*G*_*n*_,*S*) and, in consequence, the number of duplication nodes in *A*={*a*_1_,*a*_2_,…,*a*_*n*_,*v*,*root*(*G*_∗_)} in *G*_∗_ and *B*={*a*_1_,*a*_2_,…,*a*_*n*_,*v*,*root*(*G*_*n*_)} in *G*_*n*_ are equal. It follows from the properties of star S2, that in *G*_*n*_ node *v* is a speciation mapped to ⊤. Hence, all predecessors of *v* are duplications in *G*_*n*_. Thus, we have exactly *n*+1 duplications in *B*. On the other hand, by star S2, *root*(*G*_∗_) is a speciation, therefore all remaining nodes in *A* are duplications.

We conclude that *G*_*n*_ has the ⊤-cluster containing duplications from *A*, and *G*_∗_ has a cluster (mapped below ⊤) containing duplications from *B*, respectively. These two clusters we call *high clusters*. If the root of one of *T*_*i*_’s is a duplication, then it can be merged with the high cluster in both rootings. Otherwise, if every root of these subtrees is a speciation then the high cluster is disjoint with clusters from *T*_1_,*T*_2_,…,*T*_*n*+1_. Moreover, if *b* is a duplication then the high cluster contains *b* in *G*_∗_. However, in *G*_*n*_ the cluster of *b* will be disjoint with the ⊤-cluster due to the speciation node *v*. Combining the above observations we obtain our formula.

Lemma 1 and Lemma 2 complete the case of empty rootings. We proved that rooting on empty edge has the best *EC*.

### Episodes in a gene tree with a double edge

We start with two technical lemmas on the properties of the plateaus.

#### **Lemma3**.

If the DL-plateau consists of exactly one double edge then the D-plateau and the DL-plateau are equal.

#### *Proof*.

Let 〈*v,a*〉 be the DL-plateau edge (see Fig. 2). It follows from the property of star S3 that both *v* and *a* are mapped to ⊤ in the DL-minimal rooting and their children (if present) are mapped below ⊤. Hence, the root is a duplication, while *v* and *a* are speciation nodes. Now, it is easy to show that rooting on edge 〈*v,b*〉 (or 〈*v,c*〉) induces one additional gene duplication at *v*. We conclude that the only edge with the minimal duplication cost is 〈*v,a*〉.

We write that a node *g* from unrooted gene tree *G* is a *super-duplication*, if *g* is a duplication in every rooting of *G*. Please recall, that the plateau is a subtree of a gene tree, thus a leaf of the D-plateau may refer to an internal node of a gene tree. For example, in Fig. 3, the D-plateau of *G*_1_ has four leaves: one is an internal node of *G*_1_ and others, labeled *a, c, e*, are leaves of *G*_1_.

#### **Lemma4**.

If the DL-plateau has a double edge then

- every leaf of the D-plateau is a speciation in every rooting from the D-plateau,
- and every internal node of the D-plateau is a super-duplication.

#### *Proof*.

For the first part of the proof, let us assume that *v* is a leaf of the D-plateau. By using the notation from Fig. 2, let *v* be a center of a star such that 〈*v,a*〉 belongs to the D-plateau. Assume that *v* is a duplication in every D-minimal rooting. Then, the D-minimal rooting *G*_〈*v,a*〉_ has one duplication in *v*. The edge 〈*v,b*〉 does not belong to D-plateau, therefore, the rooting *G*_〈*v,b*〉_ has at least one more duplication than *G*_〈*v,a*〉_. Hence, *G*_〈*v,b*〉_ has two duplications in *v* and in the root. Moreover, the root of *G*_〈*v,a*〉_ is not a duplication. However, this is possible only when *T*_*a*_ and *T*_*v*_ are mapped below ⊤, thus the 〈*v,a*〉 is an empty edge, which is a contradiction with Theorem 2. This completes the first part of the proof.

Next, if the DL-plateau consists of exactly one double edge, then, by Lemma 3 the property holds trivially. Now, we assume that the DL-plateau has more than one edge. We show that every internal node *v* of the DL-plateau is a super-duplication. From Theorem 2 we know that *v* is incident to at least two double edges. Hence, in any rooting at least one of its children is mapped to ⊤. We conclude that *v* is a duplication mapped to ⊤.

Let us consider a path *p*=*v*_1_,*v*_2_,…,*v*_*n*_ (*n*>1) connecting an internal node *v*_1_ from the DL-plateau with a leaf *v*_*n*_ from the D-plateau. We show that the first *n*−1 nodes on *p* are duplications for every rooting placed on this path. It follows from the first part of this proof that *v*_1_ is a super-duplication mapped to ⊤. Hence, when rooting at 〈*v*_*n*−1_,*v*_*n*_〉, we have *n* gene duplications: for *v*_1_,*v*_2_,…,*v*_*n*−1_ and one for the root. All edges from *p* are elements of the D-plateau, thus moving the root to other edges on *p* will preserve the total number of gene duplications.

It should be clear that the same holds when choosing other root positions. We omit the details.

In the next lemma we show that rootings at edges of the D-plateau induce the same *EC* cost.

#### **Lemma5**.

If an unrooted gene tree *G* has no empty edge then for any D-minimal rooting of *G* denoted by *G*_∗_

$$ \mathsf{EC}(G_{*}) = \mathsf{EC}(T_{1},T_{2},\dots,T_{n}) + 1, $$

where *T*_1_,*T*_2_,…,*T*_*n*_ are the rooted subtrees of *G* obtained from *G* by removing all internal nodes of the D-plateau.

#### *Proof*.

It follows from Lemma 4 and its proof that all internal nodes of the D-plateau are present in the ⊤-cluster in the clustering with minimal number of clusters. This cluster is separated from other duplication clusters by speciation nodes located on the border of the D-plateau. Thus, the clusters induced by optimal solution to *EC* for *G*_∗_ are the clusters induced by optimal solution to *EC* of *T*_1_,*T*_2_,…,*T*_*n*_ plus the ⊤-cluster.

### Solutions

Now we present solutions to our unrooted episode clustering problem.

#### **Theorem3** (Solution to single-UEC).

For any gene tree *G*, an edge *e* is optimal for single-UEC, if either *e* is empty or *e* is in the D-plateau and *G* has a double edge.

#### *Proof*.

The first part of the proof follows immediately from Lemma 2 and the second part from Lemma 5.

#### **Theorem4**.

For a collection of unrooted gene trees *G*_1_,*G*_2_,…,*G*_*n*_, if every gene tree has a double edge then rooting every gene tree on an edge from the D-plateau yields the optimal solution for UEC.

#### *Proof*.

Assume that *n*=2 and let $G^{\prime }_{1}$ and $G^{\prime }_{2}$ be two D-plateau rootings of *G*_1_ and *G*_2_, respectively. It should be clear that *EC*(*G*1′,*G*2′)=*EC*(*T*), where *T*=(*G*1′,*G*2′). Next, by Lemma 5, *EC*(*T*) is independent on the choice of rooting of *G*_1_ and *G*_2_, as long as the rootings are in the D-plateau. Therefore, we conclude that *EC*(*T*) is the solution to UEC Problem for *G*_1_ and *G*_2_. This observation can be easily generalized by induction to any *n*.

Note that we cannot generalize the property stated in Theorem 4 to gene trees with empty edges. The example is shown in Fig. 3. Consider the dataset {*G*_1_,*G*_2_}. *G*_1_ has five D-minimal rootings, while *G*_2_ has exactly one. In *G*_2∗_ we have one ⊤-cluster, therefore *G*_2∗_ with *G*_1∗_, i.e., the empty edge rooting of *G*_1_, have two duplication clusters. However, the best clusterings for {*G*_1_,*G*_2_} having exactly one cluster are obtained for *G*_1,1_, *G*_1,2_ or *G*_1,3_. On the other hand, the best clusterings can be also obtained for empty edge rootings, e.g. {*G*_1,∗_,*G*_4,∗_} with cost 2 for the input {*G*_1_,*G*_4_}. From these examples, we see that the empty edges have different properties than double edges in the context of UEC, and we cannot generalize Theorem 4 to empty edges.

#### **Theorem5** (Candidate rootings for UEC).

For a collection of unrooted gene trees $\mathcal G$, the solution to UEC is induced by a rooting edge *e* of $G \in \mathcal G$ satisfying:

- if *G* has a double edge, then *e* is any D-minimal edge in *G*,
- if *G* has an empty edge, then *e* is an element of star S2.

#### *Proof*.

**Fig. 5:**
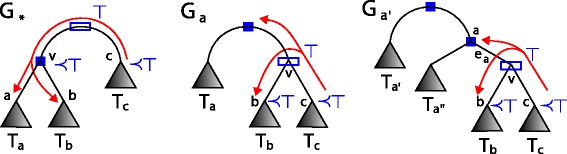
Trees from Theorem 5 and Lemma 6. The rootings of *G* from Theorem 5 and Lemma 6. We use the notation *G*_*a*_ instead of *G*_〈*v,a*〉_. See Fig. 4 for a legend of the symbols used

If some $G \in \mathcal G$ has a double edge then the property follows from Theorem 4 and Lemma 5 For gene trees with an empty edge *e*_∗_ we show that any D-minimal rooting of the edge that is not adjacent to *e*_∗_ can be equivalently replaced by a rooting adjacent to *e*_∗_. By using the notation from Fig. 2, let $T_{a}=(T_{a'},T_{a^{\prime \prime }})$ such that *a*^′^ and *a*^′′^ are the roots of $T_{a^{\prime }}$ and $T_{a^{\prime \prime }}$, respectively. We show that the rooting *G*_〈*v,a*〉_ denoted by *G*_*a*_ (see Fig. 5) has the same duplication episodes as the rooting $G_{a^{\prime }}$ obtained for the edge 〈*a,a*^′^〉. In both rootings *v* is a speciation, therefore the structure of clusters present in *T*_*b*_ and *T*_*c*_ is the same in both rootings. The edge 〈*v,a*〉 is *a*-incoming, thus the roots are duplications mapped to ⊤. From the fact that 〈*a,a*^′^〉 is in the D-plateau we have that *a* is a duplication. Thus, every root and *a* induce the ⊤-cluster. Finally, if *a*^′′^ is a duplication node, then in both rootings it will be a member of the ⊤-cluster. We proved these two adjacent rootings have the same structure of clusters. Therefore, it is sufficient to choose the rooting *G*_*a*_ instead of $G_{a^{\prime }}$. This proof can be naturally extended by induction to any edge from the D-plateau.

We conclude that for a gene tree *G* we have at most 5 candidates for rootings. For instance, *G*_4_ has two stars S2 in the D-plateau, therefore we have 5 candidate rootings: the empty edge rooting *G*_4,∗_ and the rootings of adjacent edges *G*_4,1_, *G*_4,4_, *G*_4,7_ and *G*_4,10_. Note that the clusters from *G*_4,1_ are equivalent to clusters from *G*_4,2_ and *G*_4,3_. Similar property holds for other candidates.

Next, we show that the condition U2 can be improved.

#### **Lemma6**.

Under the assumptions from Theorem 5. Let the set of clusters induced by the solution to UEC contains ⊤-cluster. Then, the condition (U2) from Theorem 5 can be refined as follows:

- if *e*_∗_ is the empty edge in *G*, then *e* is one among at most two non-adjacent edges such that *e*=〈*x,y*〉 is adjacent to *e*_∗_ and M_∗_(*x*)=M_∗_(*y*), where M_∗_ is the lca-mapping for *G*_∗_.

#### *Proof*.

Let *G* be a gene tree with an empty edge. Let *e*_*a*_ be that edge from (U2’). By using the notation from Fig. 5, we compare the rooting *G*_∗_ and *G*_〈*v,a*〉_, denoted here by *G*_*a*_. We have the following clusters in *G*_∗_: the cluster *C* that contains *c* (if *c* is a duplication) and the cluster *X* that contains *v* (it follows from the proof of Lemma 2 that *v* is a duplication node). Thus, *X*={*v*}∪*A*∪*B* where *A* and *B* denote duplications from *T*_*a*_ and *T*_*b*_, respectively. Note that *C* has the same contribution to EC in both rootings, which follows from the property that valid mappings of *C* are the same in both rootings. In *G*_*a*_, *A* is a subset of the ⊤-cluster whose contribution to EC is already incorporated (by the assumption). The node *v* is a duplication in *G*_∗_. Hence, without loss of generality we assume that *M*_∗_(*a*)=*M*_∗_(*v*), i.e., the rooting edge 〈*v,a*〉 satisfies the condition from (U2’).

We have two cases depending on whether *B* is empty. If *B* is empty then *G*_*a*_ has “better” composition of clusters than in *G*_∗_, i.e., one cluster less then in *G*_∗_ and other clusters has the same valid mappings. Otherwise, both rootings are equivalent if *M*_∗_(*b*)=*M*_∗_(*v*) (*B* in *G*_*a*_ has the same valid mappings as *X* in *G*_∗_), or again *G*_*a*_ has a better structure of clusters than *G*_∗_ if *M*_∗_(*b*)≺*M*_∗_(*v*) (valid mappings of *X* in *G*_∗_ are included in valid mappings of *B* in *G*_*a*_). Similarly, we show that *G*_*a*_ is also better than *G*_〈*v,b*〉_ (see also rootings of *G*_4_ in Fig. 3).

We proved that among three rootings from the star S2 we can choose one candidate. The second edge is obtained from the second star S2 (sharing the empty edge) if it is present in the gene tree (see Theorem 2).

From the last lemma we have at most two candidates for any gene tree from the input collection. For example, the candidate rooting *G*_4,1_ has more flexible valid mappings than *G*_4,4_, e.g. the duplication cluster of ((*c,b*),*a*) in *G*_4,1_ has larger range of possible mappings than the duplication cluster of ((*d,b*),*a*) in *G*_4,4_, while the remaining two clusters have the same locations in the species tree. Hence, for the dataset {*G*_3_,*G*_4_}, if the ⊤-cluster is present in solution to UEC, we have two candidates *G*_4,1_ and *G*_4,7_ (which is more flexible than *G*_4,10_). Note, that the clustering costs 3 is obtained by rootings *G*_3,∗_ and *G*_4,1_ (or *G*_4,2_, *G*_4,3_).



### Algorithms

Algorithm 1 presents the solution to UEC problem. The correctness of this algorithm follows from Theorem 5 and Lemma 6. Algorithm 1 has two phases. In the first phase for every gene tree a set of candidate rootings is prepared with respect to the conditions (U1) and (U2’). To find optimal rootings we use a linear time algorithm (procedure FindOptEdge) based on greedy descent method that search a double or an empty edge in a gene tree [32]. Based on condition U2’, we divide possible solutions into two categories depending on the presence of ⊤-cluster in an optimal clustering. If the ⊤-cluster is not present then every gene tree has an empty edge (in line 10). Otherwise, we check every possible variant of rooting candidates. Note that from Lemma 6, a gene tree has two candidates if and only if the gene tree has two stars S2 that are included in the D-plateau. Thus, the overall time complexity depends on the presence of such trees in the input. From this observation we conclude the following result.

#### **Theorem6**.

The time complexity of Algorithm 1 is $O(2^{k}(\sum _{i} |G_{i}| + |S|))$, where *k* is the number of input gene trees having two stars S2 that are included in the D-plateau.

Thus, from theoretical point of view UEC is fixed parameter tractable. Later we show that *k* usually represents a small fraction (up to 5 %) of the whole input. For the cases when 2^*k*^ is still too large for efficient computation, we propose Algorithm 2, in which we first solve the instance of UEC for the collection of gene trees that have a unique candidate. Clearly, if there are rootings of the whole input that have the same cost, then this cost is optimal. The overall complexity of Algorithm 2 is the same as Algorithm 1, however, for large datasets this strategy appeared to be successful after checking just one additional candidate set (in lines 2–4).



### Experiments

We performed several computational experiments on three empirical datasets.

*Guigó dataset* consists of 53 rooted gene trees from 16 Eukaryotes from [10]. This dataset was evaluated with 71 species trees from [35], known to have the total minimal duplication cost. *Génolevures* is a dataset of 4144 gene trees [33] from nine yeast genomes [36] and two species trees: one from [37] and the second one having the lowest duplication-loss cost computed by Fasturec [38]. The third dataset *TreeFam*, spanning 25 mostly animal species, consists of 1274 curated gene family trees from TreeFam v7.0 [39]. The species tree for TreeFam is based on NCBI taxonomy.

We implemented our algorithms and the algorithms for the rooted variant of EC Problem (based on [29]). In our experiments the rooting candidates were used to compare the results for UEC with the model of mappings (for rooted gene trees) proposed in [28].

We performed two series of 74 computational experiments, one for our model and one with the model described in [28]. The total running time of our program was about 7 minutes on a standard PC workstation. For every dataset we were able to find solutions to UEC by testing at most two rooted instances of input gene trees (see Algorithm 2). The summary of experiments is depicted in Table 1.

**Table 1:** Experimental results

Set	# Species trees	# Leaves	# Gene trees	*k*	Our model		Model [28]	
					*EC*	% Locations	*EC*	% Locations
Guigó	71	16	53	0	4	12,9 %	5	16,1 %
Génolevures	1 [37]	9	4144	55	17	100 %	17	100 %
	1 [38]	9	4144	156	17	100 %	17	100 %
TreeFam	1	28	1274	67	45	81,8 %	45	81,8 %

For the Guigó dataset we found four duplication clusters, while for the rooted model from [28] we located five clusters. The difference can be explained by the properties of our model that is more flexible: the input trees are unrooted and the model of valid mappings is more generic. Observe that this dataset has unique rooting candidates (*k*=0).

Génolevures is the most complex dataset due to its size and potentially large parameter *k*. Despite these properties, Algorithm 2 located 17 clusters for the filtered input with all unique rooting candidates. In other words, in this filtered dataset a duplication cluster is present in every node of the species tree. Obviously, the whole input dataset has the same property. The same holds for the model from [28].

**Fig. 6:**
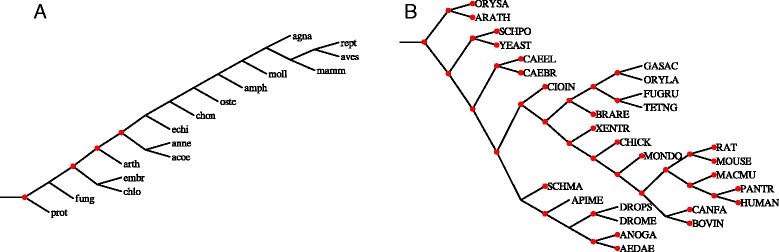
Duplication clusters in empirical datasets. Duplication clusters (*marked by red circles*) inferred from experiments. **a** Guigó species tree (chosen from 71 species trees from [35] as the most biologically reasonable [40]). **b** TreeFam species tree based on NCBI taxonomy

In TreeFam we located 45 clusters for the filtered dataset with unique rooting candidates. Then, Algorithm 2 found the solution having the same cost for the whole dataset (see Fig. 6). The same result was obtained for the model from [28] (see Table 1).

## Conclusions

In this article we presented the first solution to the open problem of the duplication episode clustering for case when the input collection is composed of unrooted gene trees. By using theoretical properties of the unrooted reconciliation we proved that the problem has nice mathematical and computational properties. From practical point of view, we were able to provide efficient algorithms and tools that were successfully applied to locate duplication clusters in real datasets.

From the computational point of view the complexity of our algorithms depends on the parameter *k*, i.e., in the worst case EC Problem has to be solved 2^*k*^ times in order to find a solution to UEC. Even if *k* usually represents a small fraction of the whole input it can be still large, e.g. *k*>100 for the yeast dataset, which may prohibit computation of all possible variants. Here we proposed a solution, that is based on the observation that the clustering induced from the input gene trees having unique candidates (that is, without *k* gene trees with non-unique variants), usually represents an optimal solution for the whole input. Thus, the strategy that we applied in Algorithm 2, i.e., first cluster easy part and then try to incorportate the hard one by using already identified clusters, appeared to be successful even for potentially complex datasets.

Our computational experiments show that the duplication clusters are usually located in large parts of the species tree especially when the input dataset consists of thousands of gene trees. To provide more detailed information on the duplication clusters, we plan to study minimal episode problem (ME) which is a natural extension of the episode clustering problem. In the future we plan to extend the episode clustering problem by using other types of valid mappings.

Our software for solving unrooted episode clustering problem is publicly available at http://www.mimuw.edu.pl/jpaszek/uec.php.

## Abbreviations

D: gene duplication
DL: Gene duplication and loss
EC: episode clustering for rooted gene trees
lca: least common ancestor
UEC: episode clustering for unrooted gene trees
